# 833. Artificial Intelligence in Burn and Wound Care: Image Analysis, Prediction, and Clinical Integration

**DOI:** 10.1093/jbcr/irag033.272

**Published:** 2026-04-06

**Authors:** Joshua Khorsandi, Justin Kahen, Liahm Blank, Joshua MacDavid

**Affiliations:** Kirk Kerkorian School of Medicine at University of Nevada, Las Vegas, NV; Kirk Kerkorian School of Medicine at University of Nevada, Las Vegas, NV; Kirk Kerkorian School of Medicine at University of Nevada, Las Vegas, NV; Department of Plastic and Reconstructive Surgery, UNLV, Las Vegas, NV

## Abstract

**Introduction:**

Burn injuries and chronic wounds represent a substantial global health burden, contributing to high morbidity, mortality, and healthcare costs. Traditional wound assessment relies heavily on subjective clinician judgment, which is prone to variability and error. Artificial intelligence (AI), particularly deep learning methods such as convolutional neural networks (CNNs), has emerged as a powerful tool to provide objective, reproducible, and scalable approaches to wound evaluation and management.

**Methods:**

This narrative review was structured around a systematic search of PubMed, Scopus, and Web of Science databases from 2015–2025, supplemented by reference screening and gray literature. Studies were organized into three domains: (1) image-based wound recognition and segmentation, (2) predictive modeling of outcomes such as healing trajectories, graft success, and infection risk, and (3) integration with telemedicine and smart technologies for remote monitoring. Comparative analysis emphasized model performance, dataset diversity, clinical usability, and regulatory or ethical considerations.

**Results:**

Results: AI-driven image analysis has demonstrated diagnostic accuracies rivaling or surpassing expert clinicians, with CNN-based segmentation achieving Dice coefficients of 90–95% and burn depth classification models attaining sensitivities above 90%. Machine learning algorithms incorporating multimodal data have predicted wound healing, infection, and amputation risk with accuracies ranging from 80–95%. Early integration with telemedicine platforms and smart dressings enables real-time, remote wound monitoring and triage, extending specialist expertise to underserved or mass-casualty settings. However, challenges remain regarding algorithmic bias (particularly across skin tones), explainability, workflow integration, and regulatory approval.

**Conclusions:**

AI represents a transformative advance in burn and complex wound care, offering objective diagnostics, prognostic insights, and expanded access through telehealth solutions. Widespread adoption will depend on rigorous validation, ethical implementation, and close collaboration between clinicians, researchers, and technology developers.

**Applicability of Research to Practice:**

By supporting earlier interventions, standardizing wound assessments, and enhancing remote monitoring, AI has the potential to improve patient outcomes, optimize resource allocation, and reduce healthcare disparities. Future progress will depend on prospective trials, multidisciplinary collaboration, and robust frameworks ensuring equity, safety, and clinician trust in AI-guided decision-making.

**Funding for the study:**

N/A.

